# Acute toluene intoxication–clinical presentation, management and prognosis: a prospective observational study

**DOI:** 10.1186/s12873-015-0039-0

**Published:** 2015-08-18

**Authors:** Carlos Rodrigo Camara-Lemarroy, René Rodríguez-Gutiérrez, Roberto Monreal-Robles, José Gerardo González-González

**Affiliations:** 1 Servicio de Neurología, Hospital Universitario “Dr. José E. González”, Universidad Autónoma de Nuevo León, Monterrey, N.L. México, Madero y Gonzalitos S/N, Monterrey, NL 64460 Mexico; 2 Departamento de Medicina Interna, Servicio de Endocrinología, Hospital Universitario “Dr. José E. González”, Universidad Autónoma de Nuevo León, Monterrey, N.L. México, Madero y Gonzalitos S/N, Monterrey, NL 64460 Mexico; 3 Knowledge and Evaluation Research Unit, Division of Endocrinology, Diabetes, Metabolism and Nutrition, Department of Medicine, Mayo Clinic, Rochester, MN 55905 USA; 4 Departamento de Medicina Interna, Hospital Universitario “Dr. José E. González”, Universidad Autónoma de Nuevo León, Monterrey, N.L. México, Madero y Gonzalitos S/N, Monterrey, NL 64460 México

**Keywords:** Toluene, Toxicity, Rhabdomyolysis, Renal tubular acidosis, Hypokalemia

## Abstract

**Background:**

Toluene is one of the most widely abused inhaled drugs due to its acute neurologic effects including euphoria and subsequent depression. However, dangerous metabolic abnormalities are associated to acute toluene intoxication. It has been previously reported that rhabdomyolysis and acute hepatorenal injury could be hallmarks of the condition, and could constitute risk factors for poor outcomes. The objective was to describe the clinical presentation, to characterize the renal and liver abnormalities, the management and prognosis associated to acute toluene intoxication.

**Methods:**

We prospectively assessed 20 patients that were admitted to a single center’s emergency department from September 2012 to June 2014 with clinical and metabolic alterations due to acute toluene intoxication.

**Results:**

The main clinical presentation consisted of weakness associated to severe hypokalemia and acidosis. Renal glomerular injury (proteinuria) is ubiquitous. Biliary tract injury (alkaline phosphatase and gamma-glutamyl transpeptidase elevations) disproportional to hepatocellular injury is common. Rhabdomyolysis occurred in 80 % of patients, probably due to hypokalemia and hypophosphatemia. There were three deaths, all female, and all associated with altered mental status, severe acidosis, hypokalemia and acute oliguric renal failure. The cause of death was in all cases due to cardiac rhythm abnormalities.

**Conclusion:**

The hallmarks of acute toluene intoxication are hypokalemic paralysis and metabolic acidosis. Liver injury and rhabdomyolysis are common. On admission, altered mental status, renal failure, severe acidemia and female gender (not significant in our study, but present in all three deaths) could be associated with a poor outcome, and patients with these characteristics should be considered to be treated in an intensive care unit.

## Background

Toluene is present in many industrial solvents, and is responsible for environmental, accidental as well as intentional intoxications. It is the most widely abused inhaled volatile drug. It is easily accessible and used in the fabrication of various products such as paints, paint thinners, glues, adhesives and cleaning products [43]. As a recreational drug, toluene is most often inhaled nasally (glue sniffing, huffing), and produces acute neurological effects such as euphoria followed by depression [5, 20]. However, almost all organs suffer some form of alteration [20]. Toluene is metabolized by cytochrome p-450 into benzoic acid and hippuric acid, these are excreted by the kidney [8, 9]. The hallmarks of toluene intoxication are distal renal tubular acidosis type 1 (RTA-1), classically described as hyperchloremic with a normal anion-gap, as well as hypokalemia and muscle paralysis [14].

In a previous case series, we retrospectively studied 22 cases of acute toluene toxicity and found that toluene inhalation is associated with various severe metabolic alterations, including RTA-1, hypokalemic paralysis and profound metabolic acidemia [12]. We also described elevations in alkaline phosphatase (ALP), and in a few patients, instances of rhabdomyolysis and proteinuria, but our data were insufficient to determine the relevance of these findings due to incomplete work-ups. No deaths were reported in that case series. In this prospective study, we sought to examine these alterations more thoroughly, to examine possible risk factors for poor outcome and the prognosis.

## Methods

We included all patients presenting with acute toluene intoxication from September 2012 to June 2014. Patients were enrolled from the Emergency Department of the University Hospital “Dr. Jose E. González” in Monterrey, Mexico after the Ethics Committee of the Medical School of the Autonomous University of Nuevo Leon approved the study. Written informed consent was obtained from each patient. A case was considered when the chief complaint (characteristic odor and confirmation of it abuse by the patient) as well as when the metabolic alterations were attributed to toluene inhalation and after alternative diagnoses were ruled out. Cases were excluded if the patient was included in the previous case series (recurrences) [12]. Each case was identified and assessed by 3 of the authors (RMR, CRCL and RRG).

### Statistical analysis

We used the computer program SPSS, version 17.0 (SPSS Inc., Chicago, Ill., USA) for statistical analyses. We used Binary Logistic Regression using categorical co-variables with Confidence Intervals (CI) of 95 % for odds ratios (OR) to analyze the risk factors associated with survivors *vs.* non-survivors. We analyzed correlations between laboratory values using the Pearson correlation coefficient, and a *p* < 0.05 was considered as statistically significant.

## Results

We identified 20 cases fulfilling criteria for acute toluene intoxication. Their clinical and demographic characteristics are shown in Table 1. Half of all patients had a previous history of drug abuse, including cocaine and marijuana, in addition to toluene inhalation. All patients or relatives self-reported their recent inhalation of toluene (paint thinner). The average time that patients had been using inhalants was 58.6 months (range: 1–260 months, self-reported).

**Table 1:** Patient clinical and demographic data

Patients	20 (100 %)
Female	8 (40 %)
Male	12 (60 %)
Age^ç^ (range)	27.4 (17–43)
Exposure (range)	58.6 (1–260)
Signs and symptoms	
Paralysis/weakness	15 (75 %)
Altered mental status	5 (25 %)
GI symptoms	4 (20 %)
Comorbidities	
DM	1(5 %)
HIV+	1(5 %)
EKG findings	
Heart Rate	119.1 ± 12.3
ST segment depression	10 (50 %)
AV block (1st degree)	6 (30 %)
U-waves	4 (20 %)
Prolonged QT	4 (20 %)

onths

*GI* gastrointestinal (nausea, vomiting and abdominal pain), *DM* diabetes mellitus, *HIV* Human immunodeficiency virus, *EKG* electrocardiographic, *AV* Atrioventricular

ç = Years

The most common presentation was muscular weakness or paralysis, followed by altered mental status and gastrointestinal complaints including nausea, vomiting and abdominal pain (Table 1). All patients were tachycardic and tachypneic on admission. A characteristic “toluene” breath was perceptible in all patients.

The relevant laboratory alterations found are shown in Table 2. Patients were severely acidotic on admission (mean plasma venous pH: 7.14 ± 0.09). Blood lactate and glucose was normal in all cases. Therefore, lactic acidosis and ketoacidosis were excluded. Mean anion gap was 16 ± 6.9 mmol/L, with 8 out of 20 patients presenting an anion gap < 12 mmol/L. Patients were also severely hypokalemic, with a mean potassium level of 2.19 mmol/L ± 1.32 (range: 1.1–4.9 mmol/L). Other electrolytes were also affected, and hyponatremia (6 cases), hyperchloremia (9 cases) and hypophosphatemia (12 cases) were quite common.

**Table 2:** Laboratory alterations associated with toluene toxicity

Laboratory test	Value (mean ± SD)	Reference-Range
Blood pH (venous)	7.14 ± 0.09 ^*^	7.35 – 7.45
HCO3 (mmol/L)	7.5 ± 4.1 ^*^	24 – 28
Anion Gap (mmol/L)	16 ± 6.9 ^*^	8 – 12
Potassium (mmol/L)	2.1 ± 1.3 ^*^	3.6 – 5.1
Sodium (mmol/L)	136.3 ± 4.3	135 – 145
Chloride (mmol/L)	109.1 ± 7.2	101 – 111
Phosphate (mg/dL)	3.3 ± 3.1	2.5 – 4.5
Calcium (mg/dL)	8.8 ± 1.2	8.4 – 10.2
Magnesium (mg/dL)	2.9 ± 0.6 ^*^	1.8 – 2.5
AST (IU/L)	61.8 ± 59 ^*^	10 – 42
ALT (IU/L)	40.4 ± 21	38 – 126
ALP (IU/L)	152.4 ± 68.9 ^*^	5 – 37
GGT (IU/L)	103.9 ± 66.9 ^*^	0.2 – 1
Total Bilirubin (mg/dL)	0.5 ± 0.2	4.0 – 11.0
WBC (K/uL)	16.9 ± 5.8 ^*^	22 – 262
CPK (U/L)	1544 ± 1864 ^*^	4.6 – 8.0
Urinary pH	6.0 ± 0.1	22 – 262
Urinary potassium (mmol/L)	34.5 ± 32.2	25 – 125
Creatinine (mg/dL)	1.7 ± 1.9 ^*^	0.6 – 1.4
24 h Urinary protein (g)	0.9 ± 0.4 ^*^	0 – 0.15

*HCO*^*3*^ bicarbonate, *AST* aspartate aminotransferase, *ALT* alanine aminotransferase, *ALP* alkaline phosphatase, *GGT* Gamma-glutamyl transpeptidase, *WBC* white blood cells, *CPK* creatinephosphokinase, *SD* standard deviation, = outside Reference-Range

^*^ = outside reference range

Serum creatine-phosphokinase (CPK) levels were elevated in 16 cases, with a mean elevation of 6 times the upper normal limit (1544 ± 1864 U/L) and reaching 6236 U/L in one patient. Liver function tests were normal, except for ALP, which was elevated in 15 patients, with a mean of 152.4 ± 68.9 (range: 67–328 IU/L). Gamma-glutamyl transpeptidase (GGT) was elevated in all patients, with a mean of 103.9 ± 66.9 (range: 45–245 IU/L). Bilirubin was normal in all cases. Serum creatinine was elevated in 5 cases. EKG abnormalities were common (Table 1). Ten out of the 15 patients with elevated ALP and GGT underwent upper abdominal ultrasound. One patient had biliary sludge, but none had biliary tract stone disease or dilatation.

On correlation analysis, chloride levels, but not potassium levels, were correlated with blood pH (0.480, *p* = 0.04). CPK, ALP and GGT levels did not correlate with pH, potassium, phosphorus, or creatinine levels, but an expected strong correlation was found between ALP and GGT (0.816, *p* = 0.004). Creatinine levels were strongly and negatively correlated with blood pH (−0.711, *p* = 0.001).

Most patients (18/20) showed some level of metabolic acidosis and hypokalemia, with established evidence of renal potassium wasting (urine potassium >20 mmol/L). Mean urine pH was inappropriately high (pH 6 ± 0.1) and in 13 cases the anion gap as measured in the urine was normal, suggesting distal RTA-1. All patients had some degree of proteinuria, but none reached nephrotic-range proteinuria in 24-h urine collection.

On admission, all patients received parenteral potassium, with doses ranging from 400 to 800 mEq/day, depending on the severity of hypokalemia. Mean hospital stay was 4.4 ± 2.1 days, and time to resolution of both acidemia and hypokalemia was 41.72 ± 20 h. All patients received in-hospital psychiatric counselling and on discharge were referred to State-sponsored addiction clinics. Five out of the 20 patients had at least one readmission (3.2 ± 2.1 months) with identical presentations.

The characteristics of the toluene-related deaths are in Table 3. All were young females who presented with altered mental status, severe acidemia, hypokalemia, rhabdomyolysis and acute oliguric renal failure. In the course of their hospitalization they developed a nodal heart rhythm and went into cardiac arrest refractory to advanced cardiopulmonary resuscitation maneuvers. The risk factors for death we identified in non-survivors *vs.* survivors were female sex, serum creatinine ≥ 2.0 mg/dL, blood (pH) ≤ 7.15 and altered mental status on admission (Table 4). However, on regression analysis, these factors did not reach statistical significance. Potassium levels or presence of altered liver function tests were not statistically different in non-survivors compared with survivors.

**Table 3:** Characteristics of deceased patients on admission

Characteristics	Patient 1	Patient 2	Patient 3
Age (years)	26	24	26
Sex	Female	Female	Female
Principal symptom	AMS	AMS	AMS
Blood pH (venous)	<6.8	7.15	<6.8
Potassium (mmol/L)	1.5	1.5	1.4
CPK (U/L)	2576	656	2676
Creatinine (mg/dL)	3.9	2.3	7.7
EKG findings	ST depression	ST depression	ST depression
	T waves	AV block	

*AMS* altered mental status, *CPK* creatinephosphokinase, *EKG* electrocardiography, *AV* atrioventricular

**Table 4:** Risk factors for death in non-survivors *vs*. survivors

Factor	Unadjusted OR (CI 95 %)	*P* value
Sex (Female)	0.60 (0.14–2.51)	0.48
pH ≤ 7.15^a^	0.37 (0.10–1.41)	0.14
Creatinine ≥ 2.0 mg/dL^a^	1.50 (0.25–8.98)	0.65
Altered mental status^a^	1.50 (0.25–8.98)	0.65

*OR* Odds ratio, *CI* Confidence Interval

^a^: On admission

## Discussion

The most common clinical presentation in acute toluene intoxication was muscular weakness due to hypokalemia (hypokalemic paralysis, a life-threatening complication that can lead to respiratory depression), a finding supported by previous reports [6, 12, 36, 45]. In contrast to the previous retrospective case series [12], we observed an alarmingly high mortality rate (15 %), and considering we gathered a similar number of patients over only 22 months, the incidence of acute toluene intoxication appears to be higher than previously recognized. Alterations in mental status were relatively infrequent, but it is worth mentioning that all of the patients who died presented with this clinical feature. Other interesting similarities between all fatal cases were female sex, severe acidemia and renal failure on admission. These patients also had rhabdomyolysis, which could partially account for oliguric renal failure.

The mechanism of hypokalemic paralysis is an increased ratio between intra- and extracellular potassium concentrations, which alters membrane polarization and thereby alters the function of excitable tissues such as nerve and muscle [2]. We found inappropriately high urinary levels of potassium in 15 patients (urine potassium >20 mmol/L), confirming that potassium wasting is another mechanism responsible for hypokalemia in toluene intoxication. Inadequate intake, gastrointestinal losses, intracellular shift and diuretic use are all common causes of hypokalemia and should be included in the differential diagnosis. Intracellular shift can be secondary to insulin excess, thyrotoxic periodic paralysis, among others. A limitation of our study is the lack of objective studies that validated the diagnosis of toluene intoxication, such as hippuric acid measurements in urine or the identification of other toluene byproducts in urine or serum this due to the fact that they were not assays available for these metabolites in our country. However, in our study, toluene intoxication diagnosis was established by a compatible clinical presentation and either the patients or relatives statement of intentional toluene inhalation. Besides toluene intoxication, other conditions may present with secondary RTA, hypokalemia and paralysis, such as Sjögren’s syndrome, hypothyroidism and systemic lupus erythematosus were discarded [15, 33, 34].

An association between distal RTA-1 and toluene inhalation was first described in 1974 [46]. Since then, the acid base abnormalities caused by toluene intoxication have been well characterized. Distal RTA-1, classically described as a hyperchloremic metabolic acidosis with hypokalemia, is characterized by an inability to lower urine pH despite severe acidemia and minimal HCO_3_ wastage. The main proposed mechanisms in toluene-induced distal RTA-1 is an inability of the distal tubule to excrete hydrogen ions as ammonium, mediated by decreased proton conductance through the active conduction pathway [7], as well as overproduction of hippuric acid by toluene metabolism [14]. Unfortunately, measurements of urinary fractional excretion of bicarbonate or urine chloride to determine urinary anion gap were not obtained. These investigations would be useful in understanding the underlying pathophysiology. Irrespective of the mechanisms involved, severe acidemia seems to be associated with a poor prognosis.

Toluene might cause both glomerular and tubular damage. Fanconi syndrome and hematuria have all been previously associated with toluene intoxication [14, 37, 41, 45]. We found that all patients showed some degree of proteinuria, which supports the hypothesis of Fanconi syndrome as characteristic of this entity, although 24 h levels of protein did not reach nephrotic-range levels. Cross-sectional studies of workers chronically exposed to toluene and other organic solvents found an increased urinary albumin excretion compared to controls [50]. Among industrial solvents, toluene exposure was found to confer the greatest risk for progression to end-stage renal disease in patients with established glomerulonephritis [29]. Toluene has also been associated to direct induction of acute tubular necrosis and acute oliguric renal failure [25]. Vomiting, dehydration, tubular injury and rhabdomyolysis are all possible causes of acute renal failure in toluene intoxication, and this complication seems to carry an ominous prognosis.

Rhabdomyolysis, occurring either by direct toluene muscular injury or prolonged immobility [45], was another abnormality found in the majority of patients (80 %). None of our patients reported periods of immobility and the elevated values of CPK we observed, suggest direct muscular injury. The major causes of rhabdomyolysis include trauma, ischemia, drugs, toxins and metabolic disorders. Electrolyte disturbances, notably hypokalemia and hypophosphatemia, are known causes of rhabdomyolysis that might play important roles in the setting of toluene intoxication. Muscular phosphate depletion in severe hypophosphatemia impairs the biochemical pathways of ATP production and alters the affinity of oxygen for haemoglobin in erythrocytes, also leading to muscle injury [18, 31].

Frank rhabdomyolysis is thought to occur mainly when serum potassium levels are < 2.0 mmol/L [18, 32]. In our series, the 12 patients presenting with a serum potassium < 2.0 mmol/L, all had elevated CPK, whereas serum potassium levels were > 2.0 mmol/L in all 4 patients with normal CPK values. Hypophosphatemia was also ubiquitous in patients with rhabdomyolysis. Acidosis itself has been proposed as a contributing factor in the development of rhabdomyolysis in hypokalemia [16, 26], but *in vivo* animal studies failed to show that either chronic or acute acidosis increases myoglobin renal toxicity [27]. Therefore, direct toluene muscular injury and electrolyte abnormalities appear to be the main causes of rhabdomyolysis in acute toluene intoxication.

Chronic toluene abuse has been associated with liver damage characterized by increases in transaminases and ALP [10, 11]. In our previous series, the most common alteration in liver function tests was an elevated ALP, but GGT levels were not measured and diagnostic imaging procedures to study the biliary tract were not done. Although there is evidence that chronic glue sniffing and severe acidosis (including RTA-1) are associated with osteomalacia and increased bone resorption ([17]; Fulop et al., 2004; [48]), it is unlikely that elevated levels of ALP are originated from bone in toluene intoxication. First of all, in both non-toluene causes of RTA-1 (Sjögren’s syndrome) and severe acidosis (diabetic ketoacidosis), ALP levels have actually been found to be decreased [21, 48]. Serum calcium levels were also normal. Moreover, we found that GGT, a more specific marker of biliary tract injury, was consistently elevated, in addition to the absence of concomitant alterations in ultrasound findings or elevations in serum total bilirubin levels. These findings suggest that toluene leads to direct biliary tract injury. Interestingly, ALP was not elevated and GGT was only mildly elevated in those patients who died. Liver injury in acute toluene intoxication appears to be mild and not to contribute to mortality.

The hepatotoxic potential of toluene has been recognized for some time. In 1971, O’Brien et al. described the case of a glue sniffer who was admitted with renal and hepatic failure and persisted with elevated ALP levels after 7 days of supportive treatment [39]. In 102 patients exposed to car paint, none showed alterations in any liver enzyme, including GGT and ALP [35]. However, animal studies have conclusively established the hepatotoxic effects of toluene inhalation. Subacute toluene exposure leads to liver balloon degeneration and liver function test abnormalities trough a variety of mechanisms, including increased oxidative stress and CYP2E1 dependent chlorzoxazone hydroxylation [38, 47]. Interestingly, in a rat study of toluene exposure in breathing air for a period of 4 weeks, the only liver abnormality found was increased ALP [42]. This is consistent with reports of increased ALP as the only liver alteration found in volatile-substance abusing street children (Olgar et al., 2008), as well as with our results.

Arrhythmia [45], ST wave changes related to hypokalemia, and even acute myocardial infarction have been described in association with toluene intoxication [13]. Severe hypokalemia decreases conduction velocity, increases automaticity and shortens the refractory period, leading to ST wave changes, atrioventricular block and cardiac arrest [19]. Chronic toluene abusers also have longer QT and corrected QT wave duration and dispersion compared to controls [4]. The development of cardiac arrhythmias is indeed the principal cause of death following inhalant abuse [22]. Severe acidosis could have contributed to refractory cardiac arrest. Cardiovascular monitoring and urgent correction of electrolytic and metabolic alterations are critical to prevent cardiac complications.

There is evidence suggesting that the female sex demonstrates distinct electrocardiographic patterns of ventricular repolarization associated with a longer rate-corrected QT interval, in both humans and animals [1]. Androgen deficiency could account for these changes, as it leads to alterations in rectifier potassium and calcium current densities in ventricular repolarization that provokes a prolongation of action potential and QTc interval [28, 30]. These hormonal differences make women more susceptible to drug-induced arrhythmia such as Long QT syndrome, Torsade de Pointes, among others [1, 30]. We speculate that this could account for the exclusively female mortality due to conduction defects we observed in this study.

The treatment of acute toluene toxicity consists in correction of the electrolytic and acid base alterations. Aggressive potassium replacement, hydration and close monitoring of the cardiac rhythm are essential [2, 3]. Respiratory depression due to paralysis is rare, and if present, it requires prompt intubation and mechanical ventilation. Management of hypokalemia should follow published guidelines [23, 49], and large doses are often needed. In our experience, the placing of a central line is essential for adequate potassium repletion.

On the basis of our findings, specific recommendations can be made. Patients should be evaluated for rhabdomyolysis. Acute oliguric renal failure should be treated aggressively and in case of nonreversal, dialysis might be promptly considered [24]. Acute liver injury does not appear to be clinically relevant. Urgent cardiologic consultation and evaluation are needed in most cases. The role of bicarbonate therapy remains unclear, and even though a specific recommendation cannot be made in severe cases its use might be considered [44]. After stabilization, great care should be placed on preventive measures, including patient education, rehabilitation and follow-up.

## Conclusions

Toluene intoxication due to intentional inhalation is a common admission diagnosis in Mexico and it can lead to death. The acute neurological symptoms are accompanied by severe metabolic alterations, as well as organ injury and dysfunction. The hallmarks of toluene intoxication are hypokalemic paralysis and metabolic acidosis. Liver injury and rhabdomyolysis are common. Altered mental state on admission, renal failure, severe acidemia and female gender could be associated with a poor outcome (not significant in our study), and patients with these characteristics should be treated in an intensive care unit. Further studies should establish an optimal treatment regimen, and great effort should be placed in preventive measures.

## References

[1] Abi-Gerges N, Philp K, Pollard C, Wakefield I, Hammond TG, Valentin JP (2004). Sex differences in ventricular repolarization: from cardiac electrophysiology to Torsades de Pointes. Fundam Clin Pharmacol.

[2] Ahlawat SK, Sachdev A (1999). Hypokalaemic paralysis. Postgrad Med J.

[3] Alkaabi JM, Mushtaq A, Al-Maskari FN, Moussa NA, Gariballa S (2010). Hypokalemic periodic paralysis: a case series, review of the literature and update of management.Eur. J Emerg Med.

[4] Alper AT, Akyol A, Hasdemir H, Nurkalem Z, Güler O, Güvenç TS, Erdinler I, Cakmak N, Eksik A, Gürkan K (2008). Glue (toluene) abuse: increased QT dispersion and relation with unexplained syncope. Inhal Toxicol..

[5] ATSDR (Agency for Toxic Substances and Disease Registry) (2000). Toxicological profile for toluene.

[6] Baskerville JR, Tichenor GA, Rosen PB (2001). Toluene induced hypokalaemia: case report and literature review. Emerg Med J.

[7] Batlle DC, Sabatini S, Kurtzman NA (1988). On the mechanism of toluene-induced renal tubular acidosis. Nephron.

[8] Benignus V (1981). Health effects of toluene: A review. Neunotoxicology.

[9] Blake RI, Coon M (1981). On the mechanism of action of cytochnome P-450. Evaluation of homolytic and heterolytic mechanisms of oxygen-oxygen bond cleavage during substrate hydroxylation by peroxides. J Biol Chem.

[10] Brautbar N, Williams J (2002). Industrial solvents and liver toxicity: risk assessment, risk factors and mechanisms.Int. J Hyg Environ Health.

[11] Broussard LA (2000). The role of the laboratory in detecting inhalant abuse.Clin. Lab Sci.

[12] Camara-Lemarroy CR, González-Moreno EI, Rodriguez-Gutierrez R, González-González JG (2012). Clinical presentation and management in acute toluene intoxication: a case series. Inhal Toxicol.

[13] Carder JR, Fuerst RS (1997). Myocardial infarction after toluene inhalation. Pediatr Emerg Care.

[14] Carlisle EJ, Donnelly SM, Vasuvattakul S, Kamel KS, Tobe S, Halperin ML (1991). Glue-sniffing and distal renal tubular acidosis: sticking to the facts.J. Am Soc Nephrol.

[15] Cherif E, Ben Hassine L, Kechaou I, Khalfallah N (2013). Hypokalemic rhabdomyolysis: an unusual presentation of Sjogren’s syndrome. BMJ Case Rep..

[16] Dhanani JA, McCarthy J, Fraser JF (2012). The dRTA-rhabdomyolysis connection. Anaesth Intensive Care..

[17] Dündaröz MR, Sarici SU, Türkbay T, Baykal B, Kocaoğlu M, Aydin HI, Gökçay E (2002). Evaluation of bone mineral density in chronic glue sniffers. Turk J Pediatr..

[18] Efstratiadis G, Voulgaridou A, Nikiforou D, Kyventidis A, Kourkouni E, Vergoulas G (2007). Rhabdomyolysis updated. Hippokratia..

[19] El-Sherif N, Turitto G (2011). Electrolyte disorders and arrhythmogenesis. Cardiol J..

[20] Faust RA. Toxicity Summary for Toluene. OAK Ridge Reservation Environmental Restoration Program, Chemical Hazard Evaluation Group Biomedical and Environmental Information Analysis Section Health Sciences Research Division. 1994. Available at: http://rais.ornl.gov/tox/profiles/toluene_c_V1.html. Accessed January 10, 2012.

[21] Fulop M, Mackay M (2004). Renal tubular acidosis, Sjögren syndrome, and bone disease. Arch Intern Med..

[22] Garriott J, Petty CS (1980). Death from inhalant abuse: toxicological and pathological evaluation of 34 cases. Clin Toxicol..

[23] Gennari FJ (1998). Hypokalemia. N Engl J Med.

[24] Gerkin RD, LoVecchio F (1998). Rapid reversal of life-threatening toluene-induced hypokalemia with hemodialysis. J Emerg Med.

[25] Gupta RK, van der Meulen J, Johny KV (1991). Oliguric acute renal failure due to glue-sniffing. Case report.Scand. J Urol Nephrol.

[26] Hanip MR, Cheong IK, Chin GL, Khalid BA (1990). Rhabdomyolysis associated with hypokalaemic periodic paralysis of renal tubular acidosis. Singapore Med J..

[27] Heyman SN, Greenbaum R, Shina A, Rosen S, Brezis M (1997). Myoglobinuric acute renal failure in the rat: a role for acidosis?. Exp Nephrol..

[28] Hreiche R, Morissette P, Turgeon J (2008). Drug-induced long QT syndrome in women: review of current evidence and remaining gaps. Gend Med.

[29] Jacob S, Héry M, Protois JC, Rossert J, Stengel B (2007). New insight into solvent-related end-stage renal disease: occupations, products and types of solvents at risk. Occup Environ Med..

[30] Jonsson MK, Vos MA, Duker G, Demolombe S, van Veen TA (2010). Gender disparity in cardiac electrophysiology: implications for cardiac safety pharmacology. Pharmacol Ther.

[31] Knochel JP, Barcenas C, Cotton JR, Fuller TJ, Haller R, Carter NW (1978). Hypophosphatemia and rhabdomyolysis. J Clin Invest..

[32] Knochel JP, Schlein EM (1972). On the mechanism of rhabdomyolysis in potassium depletion. J Clin Invest..

[33] Koul PA, Wahid A (2011). Distal renal tubular acidosis and hypokalemic paralysis in a patient with hypothyroidism. Saudi J Kidney Dis Transpl..

[34] Koul PA, Wahid A, Shah BA (2003). Systemic lupus erythematosus with distal renal tubular acidosis presenting as hypokalemic paralysis with respiratory failure. Saudi J Kidney Dis Transpl..

[35] Kurppa K, Husman K (1982). Car painters’ exposure to a mixture of organic solvents. Serum activities of liver enzymes. Scand J Work Environ Health..

[36] Meadows R, Verghese A (1996). Medical complications of glue sniffing. South Med J.

[37] Moss AH, Gabow PA, Kaehny WD, Goodman SI, Haut LL (1980). Fanconi’s syndrome and distal renal tubular acidosis after glue sniffing.Ann. Intern Med.

[38] Nedelcheva V (1996). Effects of acetone on the capacity of o-xylene and toluene to induce several forms of cytochrome P450 in rat liver. Cent Eur J Public Health..

[39] O’Brien ET, Yeoman WB, Hobby JA (1971). Hepatorenal damage from toluene in a “glue sniffer”. Br Med J..

[40] Olgar S, Oktem F, Dindar A, Kilbas A, Turkoglu UD, Cetin H, et al. Volatile solvent abuse caused glomerulopathy and tubulopathy in street children. Hum Exp. Toxicol. 2008 Jun;27(6):477-83. doi:10.1177/0960327108092292. 10.1177/0960327108092292 18784200

[41] Patel R, Benjamin J (1986). Renal disease associated with toluene inhalation. J Toxicol Clin Toxicol.

[42] Poon R, Chu I, Bjarnason S, Potvin M, Vincent R, Miller RB, Valli VE (1994). Inhalation toxicity study of methanol, toluene, and methanol/toluene mixtures in rats: effects of 28-day exposure. Toxicol Ind Health..

[43] Rosenberg NL, Kleinschmidt-DeMasters BK, Davis KA, Dreisbach JN, Hormes JT, Filley CM (1988). Toluene abuse causes diffuse central nervous system white matter changes. Ann Neurol.

[44] Sabatini S, Kurtzman NA (2009). Bicarbonate therapy in severe metabolic acidosis. J Am Soc Nephrol.

[45] Streicher HZ, Gabow PA, Moss AH, Kono D, Kaehny WD (1981). Syndromes of toluene sniffing in adults.Ann. Intern Med.

[46] Taher SM, Anderson RJ, Mcartney R, Popovitzer MM, Schrier RW (1974). Renal tubular acidosis associated with toluene “sniffing.”. NEngl. J Med.

[47] Tokunaga I, Gotohda T, Ishigami A, Kitamura O, Kubo S (2003). Toluene inhalation induced 8-hydroxy-2′-deoxyguanosine formation as the peroxidative degeneration in rat organs. Leg Med (Tokyo).

[48] Topaloglu AK, Yildizdas D, Yilmaz HL, Mungan NO, Yuksel B, Ozer G (2005). Bone calcium changes during diabetic ketoacidosis: a comparison with lactic acidosis due to volume depletion. Bone..

[49] Unwin RJ, Luft FC, Shirley DG (2011). Pathophysiology and management of hypokalemia: a clinical perspective. Nat Rev Nephrol.

[50] Voss JU, Roller M, Brinkmann E, Mangelsdorf I (2005). Nephrotoxicity of organic solvents: biomarkers for early detection. Int Arch Occup Environ Health..

